# The Microstructure, Electric, Optical and Photovoltaic Properties of BiFeO_3_ Thin Films Prepared by Low Temperature Sol–Gel Method

**DOI:** 10.3390/ma12091444

**Published:** 2019-05-03

**Authors:** Jiaxi Wang, Li Luo, Chunlong Han, Rui Yun, Xingui Tang, Yanjuan Zhu, Zhaogang Nie, Weiren Zhao, Zhechuan Feng

**Affiliations:** 1 School of Physics and Optoelectronic Engineering, Guangdong University of Technology, Guangzhou 510006, China; 18635208773@163.com (J.W.); han425@163.com (C.H.); 13570980324@163.com (R.Y.); zhuyj@gdut.edu.cn (Y.Z.); zgniegdut@163.com (Z.N.); zwrab@163.com (W.Z.); 2 School of Physical Science & Technology, Laboratory of Optoelectronic Materials & Detection Technology, Guangxi Key Laboratory for the Relativistic Astrophysics, Guangxi University, Nanning 530004, China; fengzc@gxu.edu.cn

**Keywords:** photovoltaic, bismuth ferrite, sol–gel method

## Abstract

Ferroelectrics have recently attracted attention as a candidate class of materials for use in photovoltaic devices due to their abnormal photovoltaic effect. However, the current reported efficiency is still low. Hence, it is urgent to develop narrow-band gap ferroelectric materials with strong ferroelectricity by low-temperature synthesis. In this paper, the perovskite bismuth ferrite BiFeO_3_ (BFO) thin films were fabricated on SnO_2_: F (FTO) substrates by the sol–gel method and they were rapidly annealed at 450, 500 and 550 °C, respectively. The microstructure and the chemical state’s evolution with annealing temperature were investigated by X-ray diffraction (XRD), scanning electron microscopy (SEM), Raman spectroscopy and X-ray photoelectron spectroscopy (XPS), and the relationship between the microstructure and electric, optical and photovoltaic properties were studied. The XRD, SEM and Raman results show that a pure phase BFO film with good crystallinity is obtained at a low annealing temperature of 450 °C. As the annealing temperature increases, the film becomes more uniform and has an improved crystallinity. The XPS results show that the Fe^3+^/Fe^2+^ ratio increases and the ratio of oxygen vacancies/lattice oxygen decreases with increasing annealing temperature, which results in the leakage current gradually being reduced. The band gap is reduced from 2.68 to 2.51 eV due to better crystallinity. An enhanced photovoltaic effect is observed in a 550 °C annealed BFO film with a short circuit current of 4.58 mA/cm^2^ and an open circuit voltage of 0.15 V, respectively.

## 1. Introduction

As an inexhaustible energy source for human beings, solar energy has gradually replaced some nonrenewable or polluting traditional fossil energy resources with its abundant resources, wide distribution and environmentally friendly nature. It provides a reliable and effective solution for the energy crisis and environmental protection. Photovoltaic power generation is an important way to utilize solar energy. The principle is that after the sunlight is irradiated onto the material, it generates electron–hole pairs, and a certain photovoltage is generated at both ends of the material because of the separation of the electron–hole pairs. Conventional solar cells are mainly prepared by using a p–n junction, and the built-in electric field generated by the depletion layer between the p–n junction can separate the photogenerated carriers, thereby generating a photocurrent [1,2]. Ferroelectric materials provide a new way for the research and development of solar cells by replacing the built-in electric field of the traditional p–n junction with what originates from ferroelectricity or the domain walls. The unique electric dipoles excited electric field is spread all over the ferroelectric material; this means that the electron–hole pairs excited by the light anywhere in the ferroelectric materials can contribute to the photovoltaic voltage, which can greatly improve the efficiency of solar cells [3,4]. In addition, different from traditional p–n junction solar cells, the open circuit voltage of ferroelectric materials is not limited by forbidden band width (*E_g_*), and they can even be 2 to 4 orders of magnitude higher than the forbidden bandwidth, reaching 10^3^–10^5^ V/cm. This is called the abnormal photovoltaic effect [5].

In recent years, the photovoltaic effect of ferroelectric materials, such as Pb(Zr,Ti)O_3_ [6], BaTiO_3_ [7,8] and BiFeO_3_ [9,10,11], have attracted a lot of research for photovoltaic application. Among many ferroelectric materials, BiFeO_3_ (BFO) is the most representative [12,13,14]. BFO not only has high polarization (P_r_ = 60–140 μC/cm^2^) but also has a relatively small band gap (E_g_ = 2.31–2.67 eV) [15,16], so that BFO can absorb a relatively wide solar spectrum and can obtain relatively high photoelectric conversion efficiency. Although the research on BFO ceramics is relatively mature, the leakage current of the BFO film prepared by the general process is large, and the hysteresis loop is not saturated, which restricts the practical application of the film. Therefore, various methods are being used to improve the ferroelectric and photovoltaic properties of BFO thin films. It is reported that many factors such as doping [17], annealing atmosphere [18], poling [19], electrodes [20] and substrate [21] all affect the photovoltaic performance of BFO thin films. Although the sol–gel method for the preparation of BFO films at high annealing temperatures has been reported [22,23], there is no systematic study on low-cost low-temperature synthesis. As reported in the literature [23], when the film is annealed at a high temperature above 550 °C, it will form more abundant defects and bound space charges, resulting in lower remnant polarization and larger leakage current. The low-temperature synthesis has advantages of low cost and low impurity concentration. Moreover, the relationship between photovoltaic performance and microstructure needs further demonstration.

In this paper, multilayer BFO films were prepared on SnO_2_: F (FTO) glass by the sol–gel method and rapidly annealed in air at the temperature of 450, 500 and 550 °C, respectively. Ethylene glycol was used as a solvent to achieve a precursor concentration of 0.4 mol/L. Compared to the previous film preparation process, this method can effectively reduce the number of layers of film preparation, thereby reducing the introduction of impurities. A dense BFO film with good crystallization and no impurity phase was prepared and an enhanced photovoltaic effect was observed. The microstructure and the relationship between the microstructure and electric, optical and photovoltaic properties of the BFO films were studied.

## 2. Experimental Section

In this paper, multilayer BFO films were prepared by the sol–gel method. Firstly, the ferric nitrate and bismuth nitrate were used as the solute, and the ethylene glycol (AR, Tianjin Zhiyuan Chemicals, Tianjin, China) was used as the solvent. An appropriate amount of ethylene glycol methyl (AR, Tianjin Zhiyuan Chemicals, Tianjin, China) was added in as the stabilizer to adjust the solution viscosity. In order to compensate for the loss caused by the volatilization of the Bi atom, the initial ratio of ferric nitrate (≥98.5%, Damao Chemical Regent Factory, Tianjin, China) to bismuth nitrate (≥99% Macklin, Shanghai, China) was set as 1:1.05. After stirring for 1 h, a 0.4 mol/L precursor was prepared and then stood for 72 h. Secondly, the FTO substrate was cleaned with acetone, absolute ethanol and deionized water in ultrasonic for 10 min respectively, and then dried. Thirdly, the spin-coating process was set at low speed 1500 rpm for 10 s, and then high speed 3500 rpm for 30 s. The wet film was baked at a low temperature of 80 °C for 10 min, and then at a high temperature of 300 °C for 10 min. This was repeated several times to get the required thickness of the film. At last, the BFO films were rapidly annealed at different temperature (450, 500 and 550 °C, respectively) for 15 min in an air atmosphere at a rapid annealing furnace.

The morphology and structure of the thin films were analyzed by scanning electron microscopy (SEM, S-3400N, Hitach, Tokyo, Japan) and X-ray diffractometry (XRD, D/MAX 2200 VPC, Rigaku, Tokyo, Japan). The XRD test conditions were as follows: scan rate 4°/min, step 0.02°, scan range 20°–80° and X-ray wavelength λ = 0.1542 nm. The measured XRD pattern was analyzed with the instrument’s analysis software, the JADE software package (JADE6.5, MDI, CA, USA). The Ultraviolet–Visible absorption spectra of BFO thin films were measured using the Evolution 220 UV–Visible Spectrophotometer (Thermo Fisher Scientific, Shanghai, China), and the band gaps were calculated. After the Au electrode was deposited through a shadow mask with a diameter of 0.5 mm, the leakage current was tested by Radiant Technologies’ Precision premier II (Radiant, Texas, United States). The J-V characteristic curves of the batteries were measured using a Keithley 2400 digital source meter (Tektronix, Shanghai, China) with an HTLD-4II 365 nm laser (Height-led, Shenzhen, China) as the light sources.

## 3. Results and Discussion

**Figure 1:**
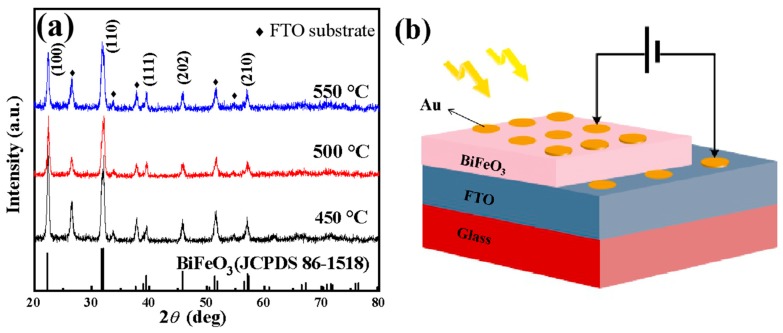
(**a**) X-ray diffractometry (XRD) patterns of BiFeO_3_ (BFO) thin films annealed at 450, 500 and 550 °C in an air atmosphere. (**b**) Sketch of the Au/BFO/FTO structure.

Figure 1a shows the XRD patterns of BFO thin films annealed at 450, 500 and 550 °C in an air atmosphere. Compared with a standard PDF card (JCPDS card no. 86-1518), the BFO films were polycrystalline with a perovskite structure and no other impurity phase except the peaks from the FTO substrate. As the annealing temperature increases, the intensity of the (110) diffraction peak becomes larger with respect to the (100) diffraction peak, while the (111), (202) and (210) diffraction peaks become weaker. The XRD results show that the BFO film exhibits a random orientation at low temperature and a highly preferred orientation at high temperature. And as the annealing temperature increases, the half width of the X-ray (110) diffraction peak becomes smaller. According to the Scherrer’s formula D=kλ/βcosθ (D is the grain size, *λ* is the X-ray wavelength, *k* is a constant and β is the half-peak width), the grain size of the BFO film becomes larger as the annealing temperature increases. Figure 1b shows the simplified graphic view of the Au/BFO/FTO structure.

**Figure 2:**
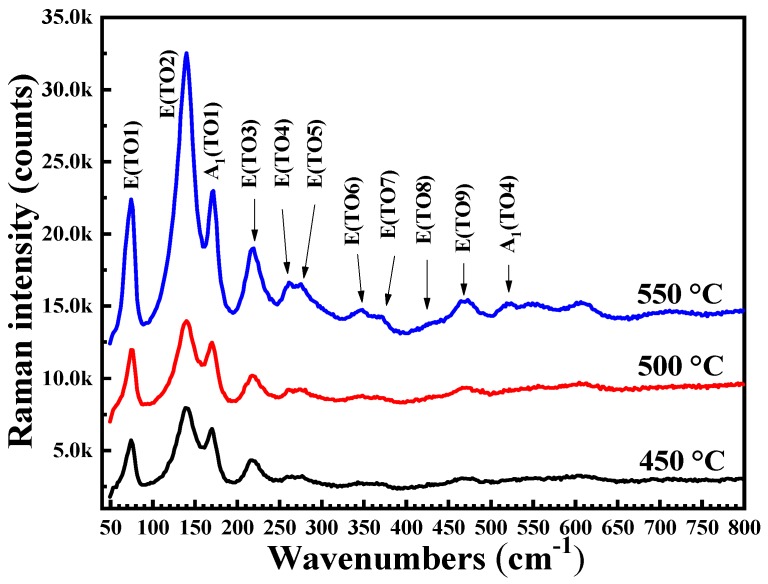
Raman spectra of BFO thin films annealed at various temperatures in an air atmosphere.

Figure 2 shows the Raman spectra of BFO thin films annealed at various temperature. All tests were performed with a 514 nm laser source at room temperature. The space group of the BFO material is R3c, and according to the group theory analysis, the BFO should have 13 Raman-active vibration modes, which is 4A1 + 9E. The optical modes of A1 and E are marked in Figure 2, wherein the peaks of 74, 138, 171, 217, 261, 275, 346, 372, 429, 470 and 520 cm^−1^ correspond to E(TO1), E(TO2), A1(TO1), E(TO3), E(TO4), E(TO5), E(TO6), E(TO7), E(TO8), E(TO9) and A1(TO4) [24,25], respectively. These values are consistent with the results calculated by the first principle based on the density functional theory [26]. Figure 2 shows that all Raman peaks are attributed to pure BFO Raman peaks and as the annealing temperature increases, the intensity of the Raman peak increases, and the number of Raman modes increases. The results show that the BFO film annealing at 550 °C has better crystallinity and is consistent with the XRD results.

**Figure 3:**
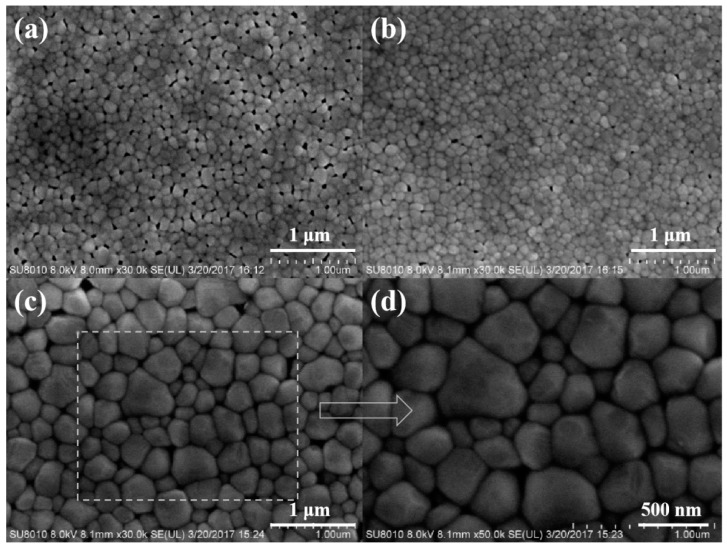
Surface morphologies of BFO thin films annealed at various temperature in an air atmosphere: (**a**) 450 °C; (**b**) 500 °C; (**c**) 550 °C; (**d**) 550 °C.

Figure 3 shows the SEM images of BFO thin films annealed at 450, 500 and 550 °C in an air atmosphere. The surface morphologies, such as film compactness, grain size, geometric shape, homogeneity and agglomeration of the nanocrystals, can be visually observed from the SEM image. Figure 3a–c are the surface image magnified 30k times of BFO films annealed at 450, 500 and 550 °C, respectively, and Figure 3d is the surface image magnified 50k times of BFO films annealed at 550 °C. It can be seen from Figure 3a–c that as the annealing temperature increases, the average grain size continues to increase, which is consistent with the XRD results of Figure 1a. After annealing at 450 °C, the average grain size was 100 nm, and after annealing at 550 °C, as shown in Figure 3d, the grain size can reach 300–700 nm. It can be seen from Figure 3 that the flatness and compactness of the BFO film are very good, and as the grain size increases, the pores on the surface of the film are also gradually reduced.

**Figure 4:**
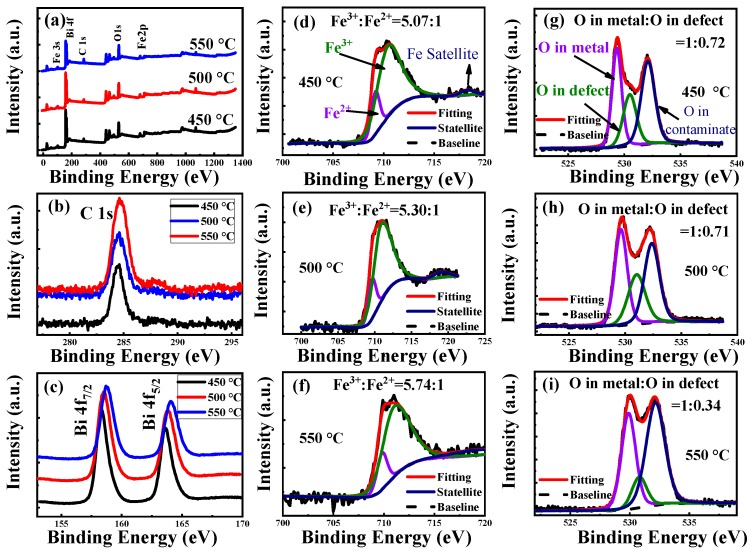
Wide-scan XPS spectra (**a**), narrow-scan XPS spectra of (**b**) C 1s, (**c**) Bi 4f of BFO films, narrow-scan XPS spectra of (**d**) Fe 2p_3/2_, 450 °C, (**e**) Fe 2p_3/2_, 500 °C, (**f**) Fe 2p_3/2_, 550 °C, (**g**) O 1s, 450 °C, (**h**) O1s, 500 °C and (**i**) O 1s, 550 °C annealed BFO films.

Figure 4a shows the wide-scan XPS spectra of the BFO films annealed at various temperatures in an air atmosphere. It can be seen that the BFO films mainly contain Fe, Bi and O elements, and there is no other impurity element, except carbon, that comes from surface contamination. Figure 4b,c shows the narrow scan XPS spectra of C 1s and Bi 4f of the BFO films annealed at various temperatures. The C 1s (284.60 eV) in Figure 4b was used as the standard to correct the binding energy of XPS spectra. The narrow spectra of Bi ions in Figure 4c shows that as the temperature increases, the content of Bi element decreases from 8.64% (450 °C) to 7.08% (550 °C) due to the volatilization of Bi^3+^. Figure 4d–f displays the narrow scan of Fe 2p_3/2_ of BFO films annealed at 450, 500 and 550 °C, respectively. It can be seen that the peaks of Fe 2p_3/2_ are asymmetrical. Because oxygen vacancies formed during growth cause some Fe^3+^ ions to become Fe^2+^ ions [23], the peaks of Fe 2p_3/2_ can be well fitted with three peaks assigned to Fe^2+^ located at 709.7 eV, Fe^3+^ located at 711.1 eV and a satellite peak located at 718.36 eV, respectively. The satellite peak demonstrates the presence of Fe^3+^ in the forms of its oxide [27]. The Fe^2+^/Fe^3+^ ratio can be calculated from the area under the deconvoluted peak of each oxidation state in the XPS spectrum [28]. The fitting results show that the Fe^3+^/Fe^2+^ ratio increases from 5.07:1 (450 °C) to 5.74:1 (550 °C). The high Fe^3+^ ions content must result in the formation of less oxygen vacancies [29], therefore the 550 °C annealed thin film containing more Fe^3+^ has less oxygen vacancies. Furthermore, Figure 4g–i displays the narrow scan of O1s of BFO films annealed at 450, 500 and 550 °C, respectively. The O 1s peaks can be well fitted with three peaks due to the lattice oxygen (O in metal) located at 529.6 eV, oxygen vacancies (O in defect) located at 531.0 eV and surface oxygen (O in contamination) located at 532.3 eV [27]. The fitting results show the relative intensity of lattice oxygen/oxygen vacancies increased obviously from 1:0.72 (450 °C) to 1:0.34 (550 °C) with the increasing annealing temperature, indicating that the oxygen vacancy is reduced under high-temperature annealing, which is consistent with the simulation results of Fe 2p_3/2_.

**Figure 5:**
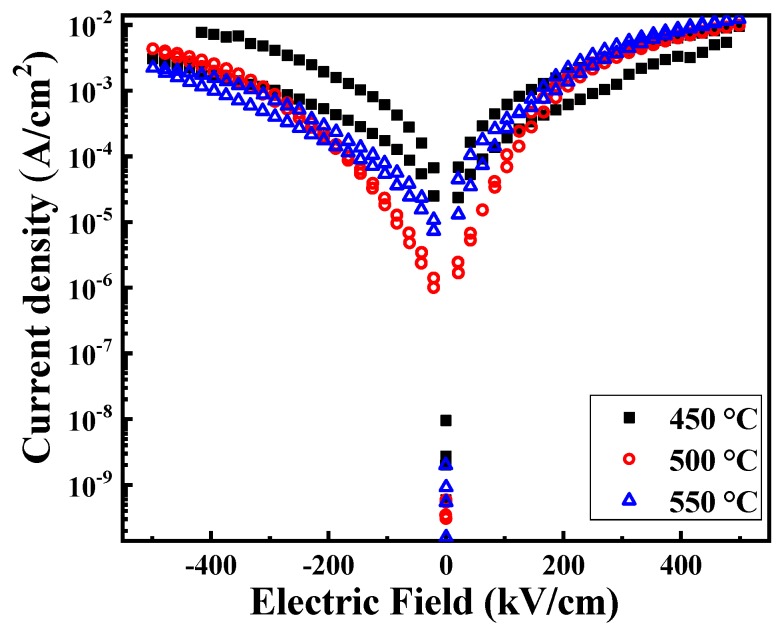
Leakage current densities of BFO thin films annealed at various temperature in an air atmosphere.

Figure 5 displays the leakage current densities of BFO thin films annealed at various temperatures in an air atmosphere. Leakage current is generated by the directional movement of the oxygen vacancies under the applied electric field. It has a great influence on the electric properties of the film. During the annealing process, the oxygen vacancies were generated due to the volatilization of Bi^3+^ and the fluctuation of the iron ion valence state [25], a relatively large leakage current will be generated when an electric field is applied to the film, as shown in Figure 5. It can be seen that after the electric field is increased to 400 kV/cm, the leakage current density of the film tends to be saturated and as the annealing temperature of the film increases, the leakage current density tends to decrease. In general, leakage current is related to phase purity, particle size and defects, such as oxygen vacancy [23,30]. It indicates that with the reduction of oxygen vacancies, improved crystallinity and increased particle size contribute to the reduction of leakage current density.

**Figure 6:**
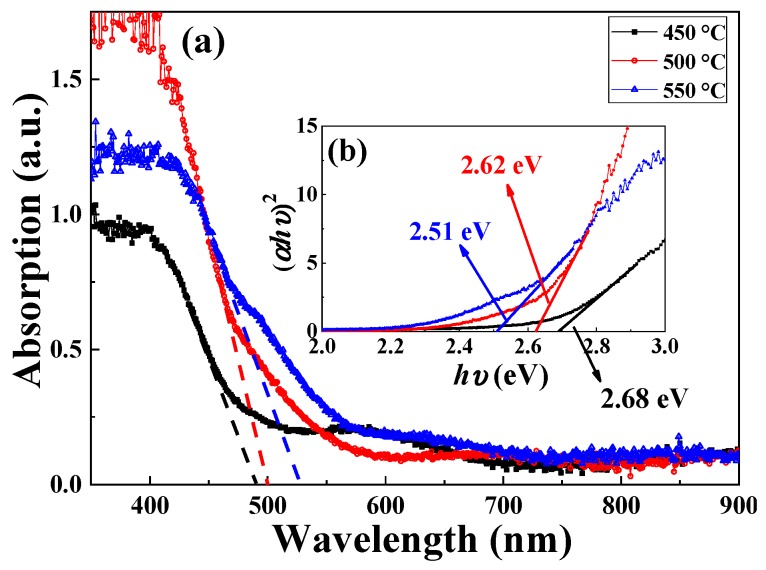
(**a**) The ultraviolet–visible absorption spectra of BFO thin films annealed at various temperature. (**b**) Plots of (*αhν*)^2^ and *hν* for the samples.

Figure 6a shows the Ultraviolet–Visible absorption spectra of BFO thin films annealed at various temperatures in an air atmosphere. At the intersection of the tangent of the absorption spectra and the x-axis, the absorption starts increasing from 494, 507 and 510 nm (2.51, 2.45 and 2.43 eV) for the samples annealed at 450, 500 and 550 °C, respectively. The absorption spectrum of the BFO film annealed at 550 °C has a wider absorption range in the visible region. In order to evaluate the optical band gaps of the BFO films, the absorption spectra are fitting based on the Tauc formula [31]:

(1) $${\left(\alpha h\nu \right)}^{2}=A\left(hv-E_{g}\right)$$

where *hν* is the photon energy, *A* is a constant, *α* is the absorption coefficient and *E_g_* is the optical band gap. Figure 6b shows plots of (*αhν*)^2^ and *hν* for the samples. The linear part of Figure 6b is directly related to the band gap, while the nonlinear part of Figure 6b corresponds to impurity absorption. From the intersection of the tangent of the linear part and the x-axis, the band gaps of the BFO films annealed at 450, 500 and 550 °C were calculated to be 2.68, 2.62 and 2.51 eV, respectively. As the annealing temperature increases, the band gap of the film becomes smaller and the absorption of visible light is enhanced. In addition, it can be found that the absorption starts increasing from lower photon energy than the optical band gap, this may be due to impurity absorption caused by impurity levels near the conduction band bottom and the valence band top. 

**Figure 7:**
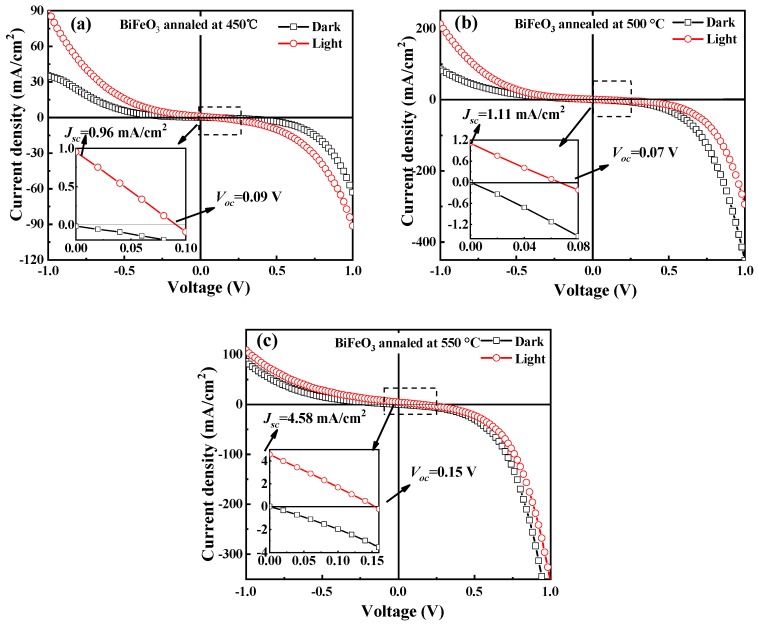
The J-V curves of BFO thin films annealed at various temperature under dark current, and 365 nm and 250 mW/cm^2^ laser lamp irradiation at (**a**) 450 °C, (**b**) 500 °C and (**c**) 550 °C.

Figure 7 shows the J-V curves of BFO films annealed at various temperatures under dark current, and exposed to a 365 nm and 250 mW/cm^2^ laser irradiation, respectively. As shown in Figure 7a–c, there are obvious differences between the photocurrent and the dark current. When the voltage is between −1 V and +1 V, the light current density is much larger than the dark current density. When the annealing temperature increases, the BFO films exhibit the enhanced photovoltaic effect. As shown in Figure 7c, the open circuit voltage and short circuit current density were 0.15 V and 4.58 mA/cm^2^, respectively. The enhancement of photovoltaic effect in 550 °C annealed BFO film is due to two reasons. On the one hand, reduced band gap and an increased absorption coefficient allow the film to absorb more photons, on the other hand, the larger polarization could separate the electron and holes more effectively [31]. In this manner, the photovoltaic effect is enhanced. Table 1 summarizes the grain size, Fe^3+^/Fe^2+^ ratio, O in metal/O in defect ratio, band gap (E_g_), short circuit photocurrent density (J_sc_) and the open circuit photovoltage (V_oc_) of BFO thin films annealed at different temperatures. 

## 4. Conclusions

In this paper, the BFO films annealed at 450, 500 and 550 °C in the air were fabricated on SnO_2_: F(FTO) conductive glass by the sol–gel method. The influence of different annealing temperatures on the microstructure, electric, optical and photovoltaic properties was studied. The XRD results show that the BFO film exhibits a random orientation at low temperature and a highly preferred orientation at high temperature. As the annealing temperature increases, the (110) diffraction peaks gradually become stronger. The SEM image shows that the grain size of the BFO film becomes larger as the temperature rises, and the pores on the surface of the film become smaller. In addition, the denseness of the film becomes better. The XPS results show that 550 °C annealed BFO thin film has a reduced oxygen vacancy concentration. The leakage current tests also show that the BFO film has smaller leakage current density after annealing at 550 °C. In addition, the Ultraviolet–Visible absorption spectrum indicates that the BFO films annealed at different temperatures have different light absorption. The band gap of the BFO film can also be adjusted by the change of annealing temperature. The band gap can be adjusted to 2.51 eV when annealing at 550 °C. The PV performance test results show that the BFO film has an enhanced photovoltaic effect after annealing at 550 °C. The open circuit voltage and short circuit current are 0.15 V and 4.58 mA/cm^2^, respectively. 

## Figures and Tables

**Table 1:** The grain size, Fe^3+^/Fe^2+^ ratio, O in metal/O in defect ratio, band gap (E_g_), short circuit photocurrent density (J_sc_) and the open circuit photovoltage (V_oc_) of BFO thin films annealed at different temperatures.

Annealing Temperature (°C)	450	500	550
Grain size (nm)	100–200	100–200	300–700
Fe^3+^:Fe^2+^	5.07:1	5.30:1	5.74:1
O in metal:O in defect	1:0.72	1:0.71	1:0.34
E_g_ (eV)	2.70	2.62	2.54
J_sc_ (mA/cm^2^)	0.96	1.11	4.58
V_oc_ (V)	0.09	0.07	0.15
